# Association of behaviour-related health risk factors with working life expectancy in adults aged ≥ 50 years: findings from the English Longitudinal Study of Ageing and the Finnish Public Sector Study

**DOI:** 10.1007/s10433-025-00896-4

**Published:** 2025-11-29

**Authors:** Katriina Heikkilä, Holendro Singh Chungkham, Jaana Pentti, Jenni Ervasti, Mika Kivimäki, Jussi Vahtera, Sari Stenholm, Paola Zaninotto

**Affiliations:** 1 Department of Public Health, University of Turku and Turku University Hospital, Turku, Finland; 2 Department of Public Health and Welfare, Finnish Institute for Health and Welfare, Helsinki, Finland; 3 Department of Epidemiology and Public Health, University College London, London, UK; 4 Psychobiology and Epidemiology Division, Department of Psychology, Stockholm University, Stockholm, Sweden; 5 Centre for Population Health Research, University of Turku and Turku University Hospital, Turku, Finland; 6 Clinicum, Faculty of Medicine, University of Helsinki, Helsinki, Finland; 7 Finnish Institute of Occupational Health, Helsinki, Finland; 8 Brain Sciences, University College London, London, UK; 9 Research Services, Turku University Hospital and University of Turku, Turku, Finland; 10 Unit of Clinical Medicine, Faculty of Medicine, University of Oulu, Oulu, Finland

**Keywords:** Working life expectancy, Health behaviours, Obesity, Smoking, Alcohol intake, Physical activity

## Abstract

**Background:**

Behaviour-related health risk factors are associated with an increased risk of early exit from the working life, but their contribution to working life expectancy (WLE) remains unclear. We investigated the associations of obesity, alcohol intake, smoking and low levels of physical activity with WLE among adults aged 50 years and older.

**Methods:**

Individuals working at study baseline with 18 years of follow-up data from the English Longitudinal Study of Ageing (ELSA) (*n* = 3233) and the Finnish Public Sector study (FPS) were included (*n* = 65,255). Obesity, alcohol consumption, smoking and low physical activity were self-reported at study baseline. WLE from age 50 to 70 years was estimated using a multi-state modelling, separately for men and women across occupational position categories (low, intermediate and high), with adjustment for age.

**Results:**

Our findings suggest that individuals who were obese, smoked, had low physical activity levels and reported heavy alcohol use (only in FPS) could expect to work fewer years than those who did not have these behaviour-related health risk factors. A higher number of risk factors was associated with shorter WLE across sex and occupational position categories in both studies. The difference in WLEs between those with no behaviour-related health risk factors and those with ≥ 2 risk factors was up to 1.5 years in ELSA and less than 1 year in FPS.

**Conclusion:**

Having multiple behaviour-related health risk factors is linked to shorter WLE after age of 50 years, a difference that may have important economic implications in societies with ageing populations.

**Supplementary Information:**

The online version contains supplementary material available at 10.1007/s10433-025-00896-4.

## Introduction

The increased old-age dependency ratio has led to many countries to put forward policy measures to extend working lives. These policy measures include increasing the statutory retirement age (van der Noordt et al. 2019; van der Mark-Reeuwijk et al. 2019; Parker et al. 2020; de Wind et al. 2018), limiting the availability of early voluntary retirement (van der Noordt et al. 2019; van der Mark-Reeuwijk et al. 2019) and restricting eligibility for disability retirement (van der Mark-Reeuwijk et al. 2019; Kadefors et al. 2019). In the UK, for example, the state pension age is expected to rise to 67 between 2026 and 2028, and in Finland, the state pension age has since 2021 been linked to life expectancy determined by birth year, starting from 65 years for those born in 1964.

Health is a key determinant of work participation and must be considered when developing policies to extend the retirement age (van der Noordt et al. 2019; van der Mark-Reeuwijk et al. 2019; Parker et al. 2020). People with a chronic disease are more likely to leave working life early due to disability retirement and unemployment (Edge et al. 2017; Shiri et al. 2020). Evidence suggests that behaviour-related health risk factors, such as overweight and obesity, excessive alcohol consumption, smoking and physical inactivity, may also play a role in shortening working lives. Findings from a meta-analysis of 25 prospective cohort studies and two nested case–control studies suggest that there is a J-shaped relationship between body mass index and disability retirement, with underweight, overweight or obese workers being more likely to experience disability retirement compared to those with normal weight (Shiri et al. 2020). The evidence for a link between alcohol consumption and the duration of working life is mixed, with positive, negative and null associations between excessive alcohol intake and early exit from working life in general or due to disability retirement or unemployment being reported (Kaila-Kangas et al. 2015; Ots et al. 2020; Hagger-Johnson et al. 2017). Findings from longitudinal studies suggest that smoking is associated with an increased risk of disability retirement and unemployment among individuals with and without a chronic disease (Ots et al. 2020; Hagger-Johnson et al. 2017). Leisure-time physical activity has been found to be associated with lower risk of disability retirement (Hagger-Johnson et al. 2017; Lahti et al. 2013), whereas no evidence for an association of physical activity with unemployment or early retirement has been found among those with a chronic disease (Ots et al. 2020). However, behaviour-related health risk factors tend to cluster (Noble et al. 2015), but the extent to which the co-occurrence of these is associated with the duration of working life is unclear.

A further limitation in the existing evidence is that most studies in this area have examined the associations of behaviour-related health risk factors and duration of working life based on data on single exit routes from employment (e.g. disability retirement, unemployment or premature death) (Shiri et al. 2021). However, a summary measure of work participation would provide more comprehensive and tangible picture of the duration of working live and its determinants over key periods in the life course. A useful summary measure for this purpose is working life expectancy (WLE), which indicates the number of years a person at a given age and with given characteristics can expect to work. Evidence points to certain behaviour-related health risk factors being linked to an increased risk of early exit from work, but the association of behaviour-related health risk factors with WLE is unclear (Shiri et al. 2021).

The aim of our study is to investigate the associations of specific behaviour-related health risk factors (obesity, high alcohol consumption, smoking and low physical activity) as well as their co-occurrence with WLE among adults aged 50 years and older, utilising data from two prospective cohort studies in England and Finland. Although behaviour-related risk factors are associated with an increased risk of several chronic conditions, such as cardiometabolic diseases, cancer and musculoskeletal disorders, the current study focuses on WLE regardless of the health status. This approach will provide policy-relevant information on the number of years individuals are likely to remain in paid employment both with and without modifiable behaviour-related risk factors.

## Methods

### Study population and data sources

Data come from the English Longitudinal Study of Ageing (ELSA) and the Finnish Public Sector study (FPS) (Supplementary Fig. 1 and Fig. 2). Initiated in 2002, ELSA is an ongoing prospective cohort study based on a nationally representative sample of adults aged 50 and older residing in private households in England (Steptoe et al. 2013). Individual- and household-level data were collected using face-to-face interviews and nurse visits, with the data periodically supplemented with clinical data obtained during visits by nurses. The response rate ranged from 55 to 82%, depending on the data collection wave (Zaninotto and Steptoe 2019). A total of 9,649 individuals aged 50+ at wave 1 (2002–2003) were included, with complete data on behaviour-related health risk factors and work participation, and who consented to having their study data linked to nationwide mortality records. We further restricted the study population to those participants who were at work at study baseline (*n* = 3233) (Supplementary Fig. 1).

FPS is a dynamic cohort study of public sector personnel in Finland. The participants were recruited from the employers’ records and followed up by record-linkage to nationwide registers for information on work participation and death (Kivimäki et al. 2007). We used data from individuals who participated in at least one data collection sweep with survey questionnaire when they were aged ≥ 50 years (2000–2002, 2004, 2008, 2011–2012, 2013–2014, 2015–2016) and had complete data on behaviour-related health risk factors, work participation and covariates (n = 65,255) (Supplementary Fig. 2).

### Ethical approvals

ELSA received ethical approval by the South Central–Berkshire Research Ethics Committee, and all participants provided written informed consent. FPS was approved by Helsinki and Uusimaa Hospital District ethics committee (HUS/1210/2016).

### Exposures

Obesity, alcohol consumption, smoking and low physical activity at baseline were ascertained from face-to-face interviews in ELSA and participant-completed questionnaires in FPS. Body mass index (BMI) was calculated using self-reported body weight and height in FPS and measured weight and height in ELSA. Obesity was defined as having a BMI ≥ 30 kg/m^2^ vs. < 30 kg/m^2^. In ELSA, high-frequency alcohol consumption was defined as drinking alcohol on 5 or more days of the week (Zaninotto et al. 2020). In FPS, heavy alcohol consumption was defined as ≥ 275 g of pure alcohol for men and ≥ 190 g pure alcohol for women per week (Kouvonen et al. 2007). High-frequency/heavy alcohol consumption was dichotomised into yes vs. no. Tobacco smoking was categorised into current vs. never or ex-smoker. In both studies, physical activity was ascertained from self-reported frequency and intensity of weekly activity and categorised into low vs. moderate-to-high activity. In ELSA, low physical activity was defined as vigorous activity on < 2 days in a week (Hamer et al. 2014). In FPS, low physical activity was defined as physical activity less than 14 metabolic equivalent (MET) hours per week (Leskinen et al. 2018). The co-occurrence of behaviour-related health risk factors (obesity, high alcohol consumption, smoking and low physical activity) was defined as the number of these behaviour-related health risk factors (0, 1, 2 or ≥ 3 risk factors). Details of the behaviour-related risk factors are provided in Supplementary Table 1.

### Outcome

In ELSA, work participation at each wave was ascertained from self-report responses to the question: “Which one of these would you say best describes your current situation?” Response options were retired, employed, self-employed, unemployed, permanently sick or disabled, looking after home or family and other. Participants were defined as working if they were employed or self-employed and not working if they were retired, unemployed, permanently sick or disabled, looking after home or family or other. Working and not working states were ascertained from waves 1 to 8 (2002–2018) and death from 2002 to 31 December 2018.

In FPS, all participants were working at baseline. Work participation during the follow-up was ascertained utilising linked data from Earnings and Accrual Register, maintained by the Finnish Centre for Pensions (Finnish Centre for Pensions 2025). Working and not working states were ascertained from dates of beginning and ending spells of employment in the Earnings and Accrual Register and death from Statistics Finland population data on deaths up to 31 December 2018. The follow-up time was aggregated into three-month periods of working or not working. When the period included time spent working as well as time spent not working, the state during that period was defined as working.

### Covariates

Age, sex and occupational position were ascertained from the employer’s records in FPS. Sex was coded as men (reference) and women in both studies. In ELSA, occupational position was ascertained from the face-to-face interview using the National Statistics Socio-economic Classification (NS-SEC) and categorised into high (NS-SEC categories 1 and 2, managerial and professional occupations), intermediate (NS-SEC category 3, e.g. administrative, non-routine sales and service occupations) and low (NS-SEC categories 4–7, routine and manual occupations, e.g. routine sales, agricultural and childcare work) (Head et al. 2019). In FPS, data on occupations were converted to International Standard Classification of Occupations (ISCO) by Statistics Finland (International Labour Organisation 2025). We categorised occupational position into high (ISCO categories 1–2, e.g. managers or physicians), intermediate (ISCO categories 3–4; skilled non-manual occupations, e.g. registered nurses) and low (ISCO categories 5–9, service and manual occupations, e.g. maintenance workers).

### Statistical analyses

WLE (from age 50 up to 70 years in ELSA and from 50 up to 68 years in FPS) was estimated from multi-state life tables approach, based on transition probabilities between three states (working, not working and dead; Supplementary Fig. 3). Participants were followed up from age 50 years up to the end of follow-up or 31 December 2018, whichever occurred first. Each participant could be in one state at a time and transition between the working and not working states, death being defined as an absorbing state. We used R packages *msm* (for multi-state survival models in panel data (Jackson 2011)) to predict transition probabilities across the three states and *elect* (utilising a Gompertz model that defines age as a time-dependent covariate (van den Hout et al. 2019)) to predict WLEs (Chungkham et al. 2023). Age in the beginning of each state was modelled as a covariate. The WLE predictions were obtained for men and women and across occupational position categories (low, intermediate and high). We estimated WLEs by individual behaviour-related health risk factor and by the number of behaviour-related health risk factors (0, 1 and ≥ 2). We calculated 95% confidence intervals for the differences in WLEs from 500 repeated simulations based on asymptotic properties of maximum likelihood estimator for the multi-state models. To examine the potential impact of differences in the baseline working status, we repeated the main analysis among all ELSA participants (*n* = 9649), including also those participants who were not working at baseline (Supplementary Table 7).

## Results

The characteristics of the participants at the start of the follow-up, at age of 50 years, are presented in Table 1. Overall, the participants in ELSA were slightly older than the participants in the FPS, and proportion of women than men was considerably higher in the FPS. In both studies, men were more likely to occupy higher occupational positions compared to women. Obesity was more prevalent among the ELSA participants, particularly among women (25% vs 17% of FPS women). In ELSA, high frequency of alcohol consumption was reported by 37% of men and 24% of women, whereas in FPS heavy alcohol consumption was reported by around 10% of men and women. Smoking was more common among women in ELSA (21%) than FPS (15%) with no marked difference among men. Low physical activity was more common among the FPS participants, with 37% of both men and women reporting low physical activity compared to 6% of men and 8% of women in ELSA, which is mostly due to different physical activity measures. In the FPS, a larger proportion of participants (both men and women) reported having no behaviour-related health risk factors compared to their ELSA counterparts. Conversely, a larger proportion of participants in ELSA than in FPS had two, three or more risk factors. Since the proportion of participants with three or more behaviour-related health risk factors were very low (2% in ELSA and 3–5% in FPS), the main results are shown for 0, 1 and 2 or more risk factors for both cohorts. In addition, results for 3 or more risk factors are only shown for FPS due to larger sample size.

**Table 1:** Participant characteristics by sex, ELSA and FPS

	ELSA (England)		FPS (Finland)	
	Men	Women	Men	Women
	(n = 1713)	(n = 1520)	(n = 13,373)	(n = 51,882)
Age, mean (SD)	57.2 (5.1)	56.3 (4.4)	54.4 (3.6)	54.1 (3.5)
*Occupational position, %*				
High	39.8	30.9	43.6	32.3
Intermediate	22.8	29.0	21.8	30.1
Low	37.4	40.1	34.6	37.6
*Behaviour-related health risk factors %*				
Obesity	22.4	24.5	18.2	16.9
Heavy drinking*	36.8	23.8	12.4	8.1
Current smoking	18.9	21.2	19.7	14.6
Low physical activity	6.4	7.6	37.3	37.4
*Number of behaviour-related health risk factors %*				
0	18.0	14.5	41.1	45.0
1	46.1	46.0	35.6	36.2
2	16.0	12.6	18.5	15.9
≥ 2	35.9	39.5	23.3	18.8
≥ 3	2.1	1.9	4.8	2.9

^*^ In ELSA high frequency of alcohol consumption and in FPS heavy alcohol consumption

Supplementary Table 2 shows the participant characteristics by occupational position. In both cohorts, behaviour-related health risk factors as well as their accumulation were more common in low and intermediate occupational positions. The most prevalent risk factors in the low occupational position group were obesity and smoking in ELSA and smoking and low physical activity in FPS. Comparison of the characteristics of the study populations and eligible populations is provided in Supplementary Table 3. In both cohorts, study populations were very similar with the eligible populations.

**Fig. 1:**
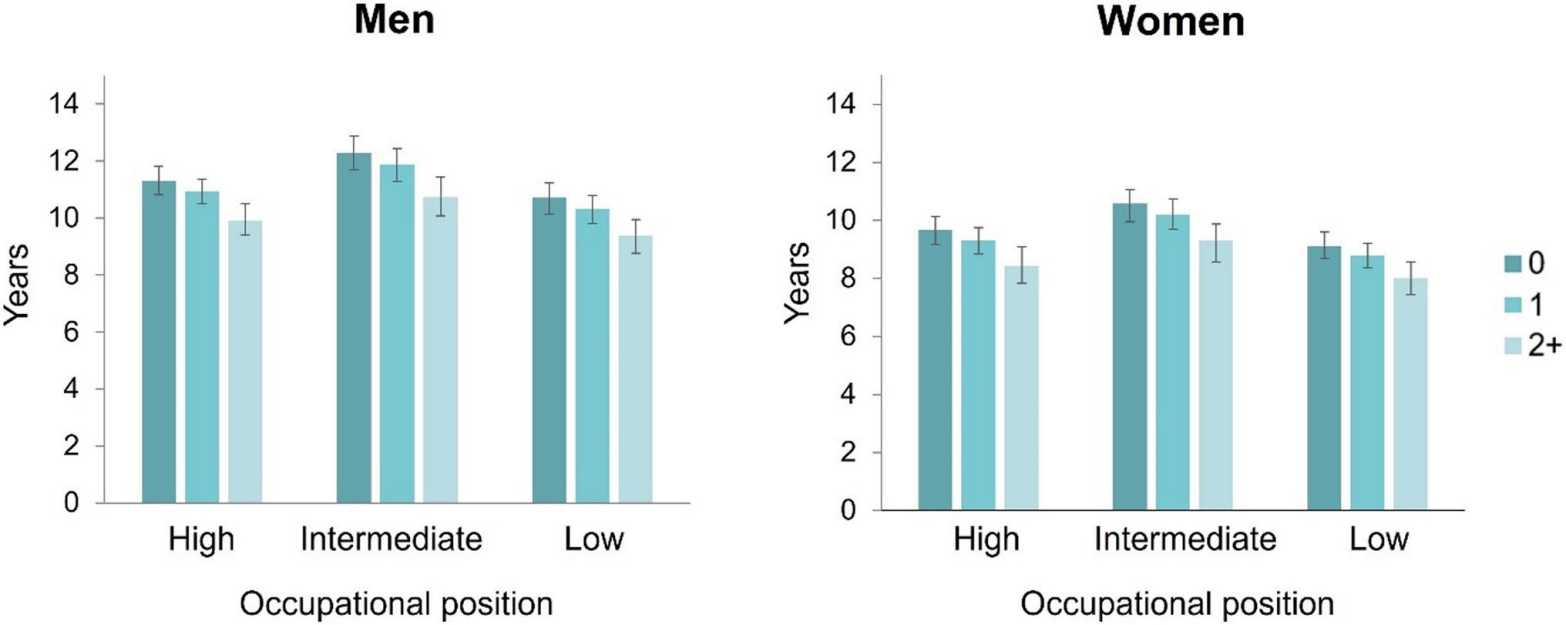
Associations of the number of behaviour-related health risk factors with working life expectancy from age 50 up to 70 years, by sex and occupational position, in the English Longitudinal Study of Ageing

**Fig. 2:**
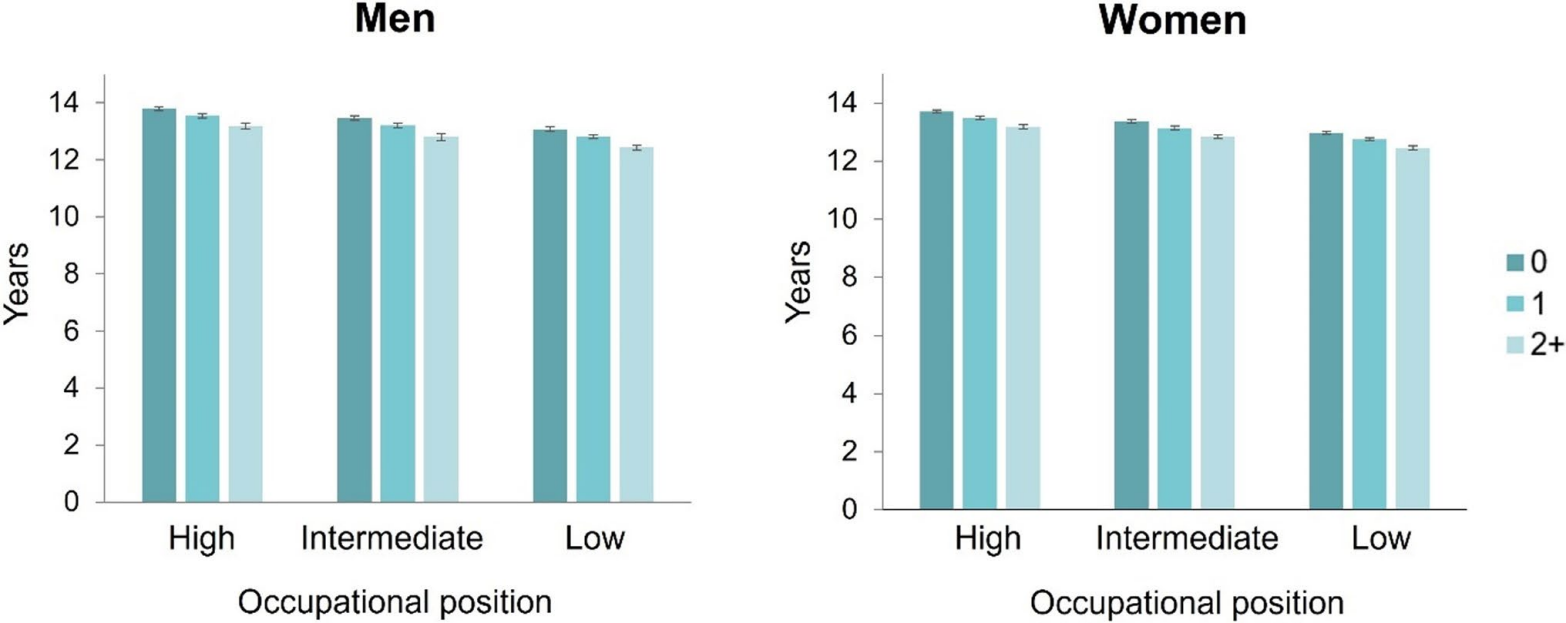
Associations of the number of behaviour-related health risk factors with working life expectancy from age 50 up to 68 years, by sex and occupational position, in the Finnish Public Sector Study

The associations of the co-occurrence of behaviour-related health risk factors with WLE, among men and women and across occupational positions, are shown in Fig. 1 for ELSA and Fig. 2 for FPS as well as in Supplementary Table 4. The increasing number of behaviour-related health risk factors was associated with shorter WLE across sex and occupational position categories in both studies. The differences in WLEs between those without and ≥ 2 more behaviour-related risk factors were about 1.4 years in men and 1.2 years for women in ELSA with no marked difference between occupational groups (Supplementary Table 5). In FPS, the differences between those without and with 2 or more behaviour-related health risk factors were smaller, 0.6 years in men and 0.5 years in women, and association with risk factors and WLE was similar across occupational position groups. When extending the comparison to those without and with 3 or more behaviour-related health risk factors, the differences in WLE increased to one year in FPS across sex and occupational position groups (Supplementary Table 4). Similar comparison was not possible in ELSA due to smaller sample size.

WLEs for individuals with and without specific behaviour-related health risk factors are shown in Table 2 and Supplementary Table 5, separately for men and women across the occupational positions. Similarly to the overall number of risk factors, individual behaviour-related risk factors (except alcohol consumption) were associated with a more marked reduction in WLE in ELSA than in FPS. Among both women and men in ELSA, low physical activity was associated with greatest reduction in WLE compared to other behaviour-related health risk factors. In addition, obesity and smoking were linked to a shorter WLE, but no difference in WLE was observed among those with and without high frequency of alcohol consumption. In FPS, obesity, heavy drinking, smoking and low physical activity were similarly associated with a reduction in WLE among women and men and across the occupational groups.

**Table 2:** Working life expectancy from age 50 up to 70 years in ELSA (England) and FPS (Finland), by specific behaviour-related health risk factors, by sex and occupational position

	Men									Women								
	High			Intermediate			Low			High			Intermediate			Low		
	WLE	95% CI		WLE	95% CI		WLE	95% CI		WLE	95% CI		WLE	95% CI		WLE	95% CI	
ELSA (England)																		
*Obesity*																		
No	11.00	10.62	11.41	11.96	11.43	12.53	10.40	9.95	10.84	9.43	9.02	9.85	10.38	9.88	10.81	8.88	8.47	9.29
Yes	10.24	9.68	10.78	11.09	10.38	11.68	9.75	9.21	10.32	8.82	8.27	9.38	9.67	7.74	10.30	9.36	7.86	8.85
Heavy drinking*																		
No	10.80	10.31	11.25	11.73	11.19	12.26	10.18	9.74	10.67	9.29	8.83	9.74	10.19	9.71	10.70	8.72	8.31	9.13
Yes	10.86	10.38	11.34	11.80	11.19	12.48	10.22	9.69	10.81	9.33	8.79	9.80	10.22	9.62	10.79	8.77	8.23	9.28
*Current smoking*																		
No	11.00	10.60	11.39	11.94	11.46	12.42	10.46	10.00	10.92	9.42	9.02	9.85	10.34	9.87	10.80	8.94	8.55	9.34
Yes	10.13	9.59	10.72	11.01	10.36	11.69	9.65	9.06	10.15	8.67	8.15	9.27	9.55	9.02	10.16	9.26	7.79	8.77
*Low physical activity*																		
No	10.94	10.52	11.33	11.95	11.43	12.46	10.39	9.97	10.81	9.40	8.97	9.86	10.33	9.88	10.85	8.90	8.51	9.29
Yes	9.40	7.95	10.19	10.26	8.37	11.44	8.90	7.61	9.78	8.07	7.05	8.99	8.92	7.74	9.84	7.76	6.63	8.46
FPS (Finland)																		
*Obesity*																		
No	13.61	13.54	13.68	13.25	13.16	13.33	12.85	12.76	12.92	13.57	13.53	13.62	13.22	13.17	13.27	12.81	12.77	12.85
Yes	13.34	13.23	13.44	12.97	12.87	13.08	12.58	12.46	12.66	13.35	13.27	13.42	12.99	12.92	13.08	12.58	12.51	12.66
Heavy drinking*																		
No	13.63	13.55	13.70	13.24	13.16	13.32	12.83	12.75	12.89	13.58	13.53	13.63	13.21	13.16	13.25	12.78	12.74	12.82
Yes	13.31	13.19	13.41	12.91	12.77	13.02	12.49	12.35	12.60	13.30	13.21	13.39	12.92	12.82	13.02	12.50	12.40	12.60
*Current smoking*																		
No	13.66	13.59	13.73	13.32	13.23	13.40	12.93	12.85	13.00	13.58	13.54	13.63	13.24	13.19	13.29	12.85	12.80	12.89
Yes	13.16	13.04	13.27	12.79	12.66	12.90	12.43	12.31	12.53	13.19	13.11	13.28	12.85	12.77	12.92	12.46	12.38	12.53
*Low physical activity*																		
No	13.69	13.63	13.76	13.33	13.25	13.41	12.93	12.85	13.01	13.65	13.60	13.70	13.29	13.24	13.34	12.89	12.83	12.93
Yes	13.38	13.29	13.45	13.00	12.91	13.10	12.60	12.51	12.69	13.37	13.31	13.42	13.01	12.95	13.07	12.60	12.54	12.65

^*^ In ELSA high frequency of alcohol consumption and in FPS heavy alcohol consumption

Finally, the findings concerning the full ELSA sample, i.e. including also participants who were not working at baseline, are shown in Supplementary Tables 6 and 7. Overall, the estimated WLEs were shorter in the total population than those estimated for the ELSA participants who were at work at baseline. In a similar vein, the estimated WLEs by single behaviour-related health risk factors were lower among the full ELSA sample compared to those participants who worked at baseline, among women and men and across the occupational positions.

## Discussion

Our findings, based on data from women and men aged ≥ 50 years in two cohort studies in England and Finland, suggest that individuals who were obese, smoked and had low levels of physical activity could expect to work fewer years than those who did not have these behaviour-related health risk factors. Having several behaviour-related health risk factors, compared to none, was also associated with a shorter working life expectancy. Overall, these patterns were similar in direction among men and women and across occupational positions, but their magnitude differed. Participants with multiple risk factors could expect to work, on average, up to 1.5 years less in ELSA (and up to 1 year in FPS) than those without: the magnitude of the reduction is modest but may have significant financial, social and health implications for the individual worker as well as economic consequences for the wider society, in terms of loss of income, reduced social participation and, particularly in Finland with an occupational health care system for employees, reduced access to health care.

Findings from previous studies point to obesity and smoking being consistently associated with increased risks of disability retirement and unemployment (Ots et al. 2020; Shiri et al. 2020; Kaila-Kangas et al. 2015; Hagger-Johnson et al. 2017), whereas the evidence for an association between alcohol consumption and physical activity with an early exit from working life is less consistent (Hagger-Johnson et al. 2017; Ots et al. 2020; Kaila-Kangas et al. 2015; Lahti et al. 2013). However, as previous studies have investigated the associations of behaviour-related health risk factors with specific exit routes from work, rather than overall working life expectancy, their results are not directly comparable to ours.

In ELSA, the behaviour-related health risk factor that was most markedly associated with a shorter working life expectancy in both men and women was low physical activity, but also obesity and smoking were linked to reduced working life expectancy. Same risk factors were also prominent in FPS with smoking being the leading risk factor for shorter working life expectancy especially in men. In terms of alcohol consumption, heavy drinking was associated with shorter working life expectancy in FPS, but we observed no difference in WLE among those with and without high frequency of alcohol use in ELSA. However, detailed comparison of the role of different risk factors for working life expectancy in ELSA and FPS is challenging, especially for alcohol consumption and physical activity, due to differences in measurement as well as cross-national variation in these behaviours.

Our findings suggest that individual behaviour-related health risk factors as well as the overall number of risk factors are linked to a reduced working life expectancy among adults aged 50 years and older. They also indicate that like many health and social outcomes, estimated working life expectancy is socioeconomically patterned, with women and men with the largest number of behaviour-related health risk factors and in the lowest occupational positions faring the worst in terms of the time they could expect to continue working. However, we did not observe differences in the association between behaviour-related health risk factors and working life expectancy across occupational groups, suggesting that both behaviour-related health risk factors and occupational position independently contribute to the length of working life. One interpretation of our findings is that whilst maintaining normal weight, reducing alcohol intake, stopping smoking and engaging in physical activity will undoubtedly improve an individual’s health and well-being, they could also support individuals in continuing to work, which in turn may have positive health, social and economic impacts in later life.

### Policy implications

The findings from our study highlight the potential impact of behaviour-related health risk factors on working life expectancy among older adults, raising important considerations for policy development. Whilst it is well established that health behaviours such as smoking, excessive alcohol consumption and physical inactivity, as well as obesity, negatively influence health outcomes, our study underscores their specific role in reducing the number of years individuals can remain active in the workforce. This has important implications not only for individuals but also for the broader economy, as a shorter working life expectancy can strain pension systems and reduce productivity. Policy interventions must therefore address these risk factors through comprehensive strategies that go beyond mere health promotion. For example, workplace wellness programmes could be tailored to support behaviour change, particularly among those in lower occupational positions who are disproportionately affected.

### Strengths and limitations

Key strengths were that we used longitudinal data from England and Finland, based on a population-representative sample and occupational cohort, respectively. In FPS, working life expectancy was estimated utilising data from a nationwide register. Furthermore, multi-state life tables allow for the modelling of transitions between multiple states (e.g. working, non-working, death) which captures the reality that individuals can move in and out of different states throughout their lives, providing a more realistic estimate of working life expectancy compared to static models.

A limitation of our study is the reliance on self-reported behaviour-related health risk factors, with the exception of measured weight and height from ELSA. Work participation was also self-reported in ELSA. Self-report data can be prone to biases, such as underreporting of behaviours like alcohol consumption and smoking, which could affect the accuracy of our findings. Additionally, despite our efforts to harmonise the exposure measures across different data sets, achieving complete comparability was challenging especially for alcohol consumption and physical activity; despite this, the patterns were mainly similar in the two countries.

In conclusion, our findings in two prospective cohort studies in England and Finland suggest that obesity, smoking, physical inactivity and excessive alcohol consumption in the Finnish cohort, as well as the co-occurrence of these behaviour-related health risk factors, are linked to approximately ~ 1 and 1.5 year shorter working life expectancies at and after age of 50 years. This difference has significant economic implications for ageing populations and society.

## Supplementary Information

Below is the link to the electronic supplementary material.

Supplementary file1 (PDF 588 KB)

## Data Availability

ELSA data can be accessed upon registration to the UK Data Service (https:/ukdataservice.ac.uk). For the FPS, the pseudonymised questionnaire data used in this study can be made available upon request to the study team. Linked register data on working participation and death require separate permission from the Findata, the Health and Social Data Permit Authority in Finland.
