# Supplementary material for: Association of behaviour-related health risk factors with working life expectancy in adults aged ≥ 50 years: findings from the English Longitudinal Study of Ageing and the Finnish Public Sector Study
Source: Eur J Ageing. 2025 Nov 29;22(1):61. doi: 10.1007/s10433-025-00896-4 (PMC12682727; doi:10.1007/s10433-025-00896-4)
Supplement: Supplementary file 1 — Supplementary file1 (PDF 588 KB) [file 10433_2025_896_MOESM1_ESM.pdf]

Online supplement

Supplementary Figure S1. Participant flow chart for English Longitudinal Study of Ageing.

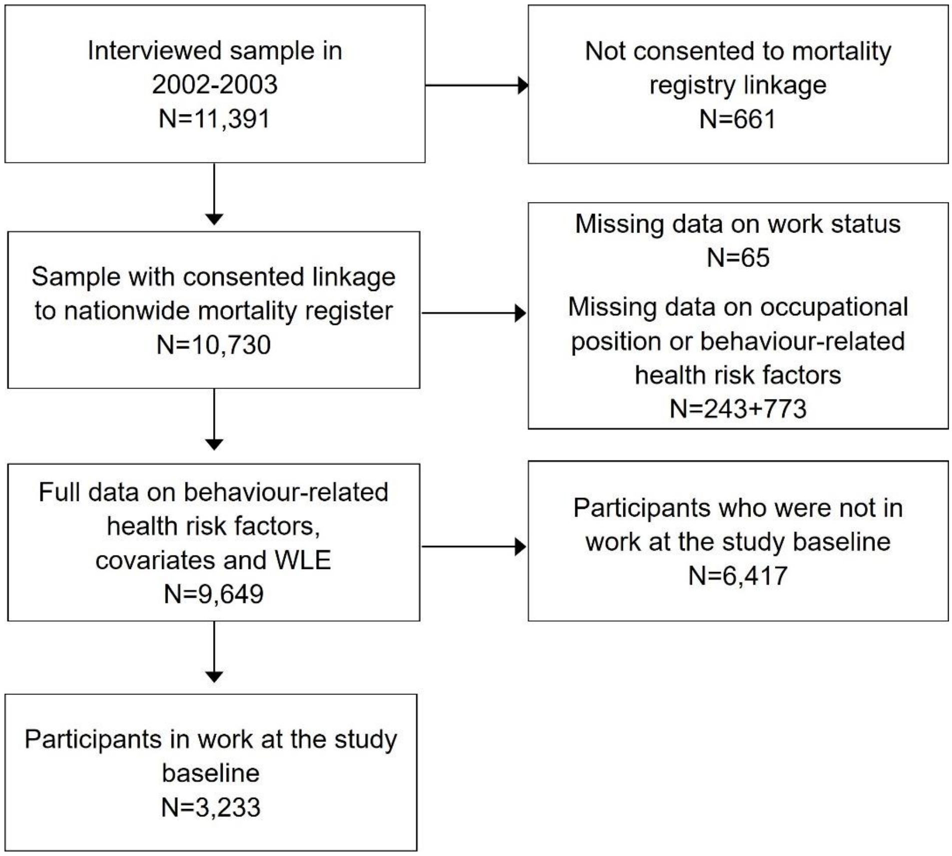

Supplementary Figure 2. Participant flow chart for Finnish Public Sector study

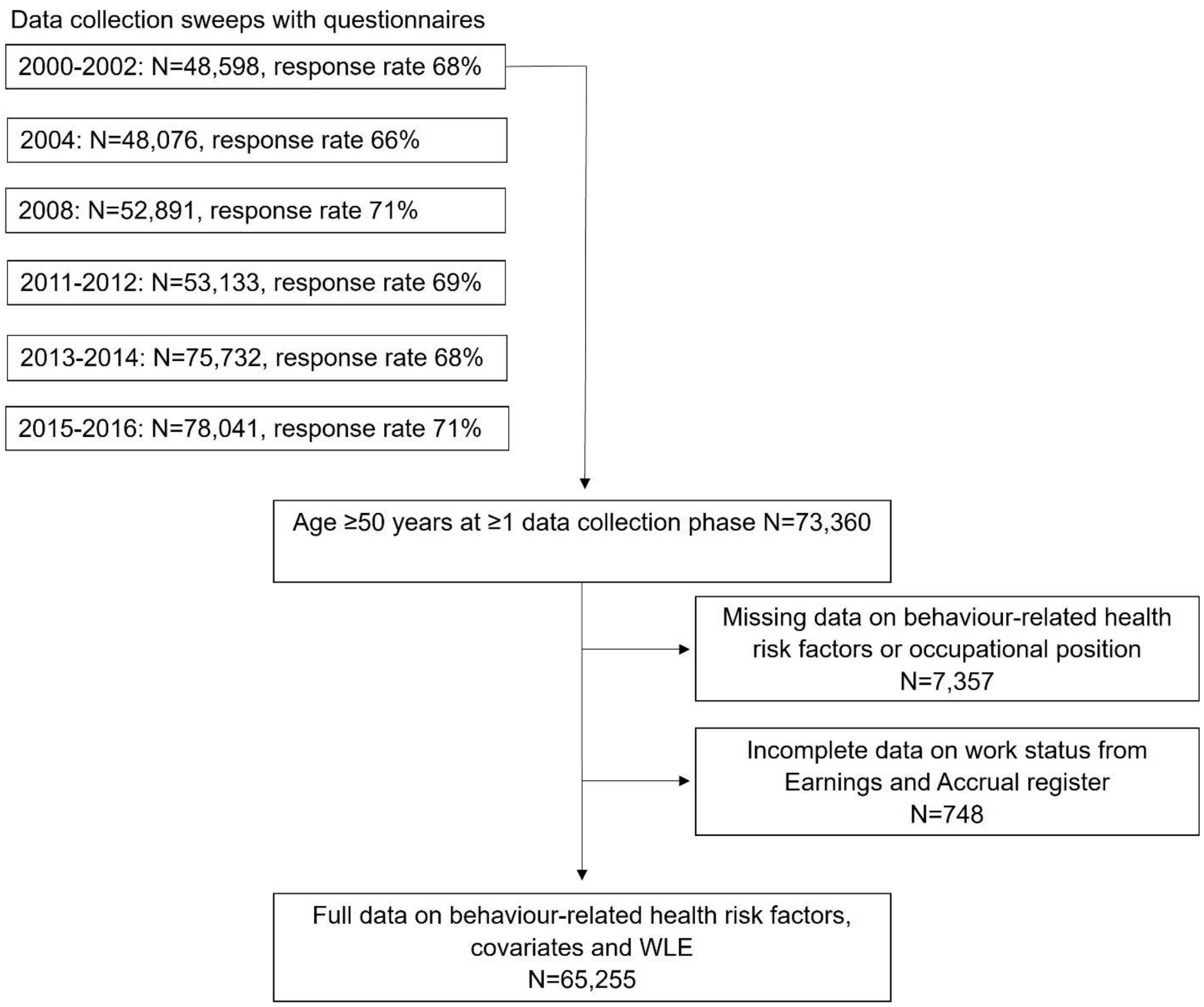

Supplementary Figure 3. WLE model transitions

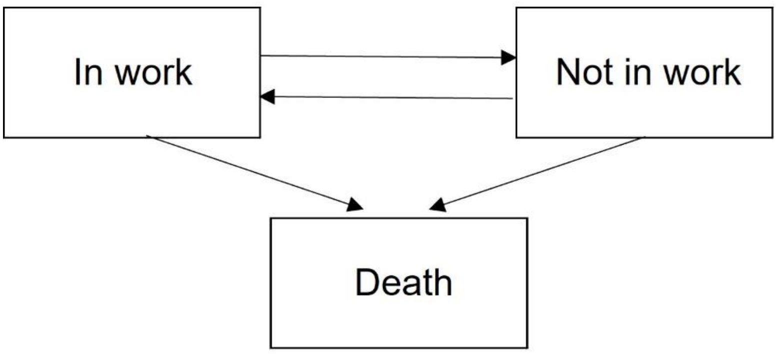

Supplementary Table 1. Measurement of the behaviour-related health risk factors in ELSA and FPS

	ELSA (England)	FPS (Finland)
**Obesity**	Body weight and height measured by study nurse.<br><br>BMI was calculated as body weight divided by square of body height.<br><br>Yes: BMI ≥ 30 kg/m^2<br>No: BMI < 30 kg/m^2	Q: “How tall are you?” / “How much do you weigh?”<br><br>BMI was calculated as body weight divided by square of body height.<br><br>Yes: BMI ≥ 30 kg/m^2<br>No: BMI < 30 kg/m^2
**Alcohol consumption**	Q: “Thinking now about all kinds of drinks, how often have you had an alcoholic drink of any kind during the last 12 months?”<br><br>High frequency of alcohol consumption:<br>Yes: drinking alcohol on 5 or more days of the week.<br>No: drinking alcohol on <5 days a week<br><br>Zaninotto et al. 2020 Sci Rep	Q: “How much alcohol (beer, wine and spirits) do you typically consume?” Based on responses of weekly alcohol units for beer, wine and spirits, weekly grams of alcohol was calculated.<br><br>Heavy alcohol consumption:<br>Yes: ≥275 g of pure alcohol for men and ≥190 g pure alcohol for women per week<br>No: <275 g of pure alcohol for men and <190 g pure alcohol for women per week<br><br>Kouvonen et al. 2007 J Occup Environ Med
**Current smoking**	Q: “Do you smoke cigarettes at all nowadays?”<br><br>Yes: Current smoker<br>No: Never and ex-smoker	Q: “Do you smoke or have you previously smoked regularly, that is daily or nearly daily?” Followed by a question “Do you still smoke regularly?”<br><br>Yes: Subjects who responded “yes” to both questions<br>No: Subjects who responded “no” to the latter question
**Low physical activity**	Q: “We would like to know the type and amount of physical activity involved in your daily life. How often you take part in sports or activities that are vigorous, moderate or light?”<br><br>Yes: Takes part in moderate of vigorous activity 3 times a months or more<br>No: No moderate or vigorous activity on a weekly basis<br><br>Hamer et al. 2014 Br J Sports Med	Q: “Estimate the average weekly hours of leisure-time physical activity (including commuting) within the previous year in walking, brisk walking, jogging and running, or their equivalent activities”<br><br>The time spent on activity at each intensity level in hours per week was multiplied by the average energy expenditure of each activity, expressed in metabolic equivalent (MET). Low physical activity was defined as weekly physical activity was less than 14 MET hours per week.<br><br>Yes: Weekly physical activity < 14 MET hours per week<br>No: Weekly physical activity ≥ 14 MET hours per week<br><br>Leskinen et al. 2018 Prev Med

References

Hamer, M., K. L. Lavoie, and S. L. Bacon. 2014. 'Taking up physical activity in later life and healthy ageing: the English longitudinal study of ageing', British journal of sports medicine, 48: 239-43.

Kouvonen, A., M. Kivimäki, A. Väänänen, T. Heponiemi, M. Elovainio, L. Ala-Mursula, M. Virtanen, J. Pentti, A. Linna, and J. Vahtera. 2007. Job strain and adverse health behaviors: the Finnish Public Sector Study, J Occup Environ Med, 49: 68-74.

Leskinen, T., S. Stenholm, O. J. Heinonen, A. Pulakka, V. Aalto, M. Kivimaki, and J. Vahtera. 2018. Change in physical activity and accumulation of cardiometabolic risk factors, Prev Med, 112: 31-37.

Zaninotto, P., J. Head, and A. Steptoe. 2020. Behavioural risk factors and healthy life expectancy: evidence from two longitudinal studies of ageing in England and the US, Sci Rep, 10: 6955.

Supplementary Table 2. Participant characteristics by occupational position, ELSA and FPS

	ELSA (England)			FPS (Finland)		
	High (n=1152)	Intermediate (n=831)	Low (n=1250)	High (n=22585)	Intermediate (n=18552)	Low (n=24118)
%	35.6	25.7	38.7	34.6	28.4	37.0
**Age**, mean (SD)	56.2 (4.2)	57.5 (5.7)	56.9 (4.5)	54.2 (3.6)	53.9 (3.5)	54.3 (3.5)
**Behaviour-related health risk factors** %						
Obesity	20.8	21.8	27.0	13.6	17.8	20.1
Heavy drinking*	40.1	30.8	21.9	11.9	8.5	6.6
Current smoking	15.2	18.4	25.4	9.7	15.8	21.1
Low physical activity	5.6	8.2	7.5	33.6	37.6	40.8
**Number of behaviour-related health risk factors** %						
0	15.4	15.9	17.7	49.4	44.4	39.1
1	48.4	45.4	44.3	35.3	35.3	37.5
≥2	36.2	38.8	38.0	15.4	20.3	23.4

Notes: * In ELSA high frequency of alcohol consumption and in FPS heavy alcohol consumption.

Supplementary Table 3. Characteristics of the study population and eligible population (workers at baseline) in ELSA and FPS.

	ELSA (England)		FPS (Finland)	
	Study population	Eligible population	Study population	Eligible population
**N**	3,233	3,607	65,255	73,360
**Age**, mean (SD)	56.8 (4.8)	56.4 (SD 4.8)	54.1 (3.5)	54.2 (3.6)
**Sex** %				
Men	53.0	52.8	20.5	20.2
Women	47.0	47.2	79.5	79.8
**Occupation** %				
High	35.6	35.7	34.6	33.7
Intermediate	25.7	27.6	28.4	28.8
Low	38.6	38.7	37.0	37.5

Supplementary Table 4. Working life expectancy at age ≥50 years by sex and occupational position.

Number of behaviour-related health risk factors	Men									Women								
	High			Intermediate			Low			High			Intermediate			Low		
	WLE	95% CI		WLE	95% CI		WLE	95% CI		WLE	95% CI		WLE	95% CI		WLE	95% CI	
**ELSA (England)**																		
0	11.30	10.82	11.83	12.27	11.68	12.87	10.72	10.14	11.22	9.66	9.15	10.13	10.57	9.94	11.07	9.12	8.68	9.59
1	10.94	10.50	11.35	11.88	11.29	12.45	10.31	9.80	10.80	9.31	8.83	9.74	10.20	9.68	10.72	8.78	8.36	9.20
≥2	9.91	9.41	10.50	10.75	10.08	11.43	9.39	8.76	9.94	8.43	7.83	9.09	9.30	8.55	9.87	8.00	7.43	8.56
**FPS (Finland)**																		
0	13.80	13.73	13.87	13.46	13.39	13.55	13.07	12.99	13.15	13.71	13.66	13.77	13.37	13.31	13.43	12.97	12.92	13.02
1	13.55	13.48	13.62	13.20	13.12	13.28	12.82	12.74	12.89	13.48	13.43	13.54	13.14	13.08	13.20	12.74	12.69	12.80
≥2	13.17	13.07	13.27	12.81	12.68	12.92	12.44	12.33	12.53	13.18	13.10	13.25	12.84	12.77	12.90	12.45	12.38	12.52
0	13.81	13.73	13.88	13.47	13.38	13.55	13.08	12.99	13.16	13.71	13.66	13.77	13.37	13.31	13.42	12.97	12.92	13.02
1	13.56	13.49	13.62	13.21	13.12	13.29	12.82	12.74	12.90	13.48	13.43	13.53	13.14	13.08	13.20	12.74	12.68	12.80
2	13.27	13.16	13.36	12.91	12.78	13.02	12.53	12.41	12.63	13.27	13.18	13.35	12.92	12.84	13.00	12.53	12.45	12.61
≥3	12.72	12.52	12.90	12.37	12.18	12.56	12.00	11.80	12.16	12.75	12.59	12.90	12.41	12.27	12.58	12.02	11.86	12.16

Notes: WLE: working life expectancy

Supplementary Table 5. Differences in working life expectancy at age ≥50 years by sex and occupational position.

	ELSA (England)			FPS (Finland)		
**Comparison**	**WLE**	**95%CI**		**WLE**	**95%CI**	
Men, Professional						
0 vs. ≥2 risk factors	1.39	0.65	2.13	0.63	0.50	0.76
Obesity	0.76	0.08	1.44	0.27	0.14	0.39
Heavy drinking*	-0.06	-0.73	0.61	0.32	0.19	0.46
Current smoking	0.87	0.18	1.56	0.50	0.37	0.63
Low physical activity	1.54	0.35	2.73	0.32	0.21	0.42
Men, Intermediate						
0 vs. ≥2 risk factors	1.52	0.62	2.42	0.65	0.50	0.79
Obesity	0.87	0.02	1.72	0.27	0.14	0.41
Heavy drinking*	-0.07	-0.91	0.77	0.34	0.19	0.49
Current smoking	0.93	0.11	1.75	0.52	0.37	0.67
Low physical activity	1.69	0.07	3.31	0.33	0.20	0.45
Men, Low						
0 vs. ≥2 risk factors	1.33	0.53	2.13	0.64	0.51	0.76
Obesity	0.65	-0.06	1.36	0.27	0.14	0.40
Heavy drinking*	-0.04	-0.77	0.69	0.33	0.19	0.48
Current smoking	0.81	0.10	1.52	0.50	0.37	0.64
Low physical activity	1.49	0.33	2.65	0.33	0.21	0.44
Women, Professional						
0 vs. ≥2 risk factors	1.23	0.43	2.03	0.53	0.43	0.62
Obesity	0.61	-0.08	1.30	0.23	0.14	0.32
Heavy drinking*	-0.04	-0.72	0.64	0.28	0.18	0.38
Current smoking	0.75	0.05	1.45	0.39	0.30	0.49
Low physical activity	1.33	0.26	2.40	0.28	0.21	0.36
Women, Intermediate						
0 vs. ≥2 risk factors	1.27	0.40	2.14	0.53	0.44	0.62
Obesity	0.71	-0.65	2.07	0.23	0.13	0.32
Heavy drinking*	-0.03	-0.80	0.74	0.29	0.17	0.40
Current smoking	0.79	0.05	1.53	0.40	0.30	0.49
Low physical activity	1.41	0.25	2.57	0.28	0.21	0.36
Women, Low						
0 vs. ≥2 risk factors	1.12	0.39	1.85	0.53	0.44	0.61
Obesity	-0.48	-1.12	0.16	0.23	0.14	0.31
Heavy drinking*	-0.05	-0.72	0.62	0.29	0.18	0.39
Current smoking	0.68	0.05	1.31	0.39	0.30	0.47
Low physical activity	1.14	0.15	2.13	0.29	0.21	0.36

Notes: Estimates adjusted for age. * In ELSA high frequency of alcohol consumption and in FPS heavy alcohol consumption.

Supplementary Table 6. Characteristics of all ELSA participants (n=9,649).

	Men (n = 4484)	Women (n = 5165)
**Age**, mean (SD)	61.6 (6.9)	61.5 (7.0)
**Work status**, %		
In work	38.2	29.4
Not in work	61.8	70.6
**Occupational position**, %		
High	37.0	24.1
Intermediate	19.4	28.4
Low	43.6	47.5
**Behaviour-related health risk factors** %		
Obesity	23.1	28.2
Heavy drinking*	35.7	21.9
Current smoking	17.6	18.3
Low physical activity	14.2	19.3
**Number of behaviour-related health risk factors** %		
0	34.1	35.5
1	44.6	44.2
≥2	21.3	20.4

Notes: Estimates adjusted for age. * In ELSA high frequency of alcohol consumption and in FPS heavy alcohol consumption.

Supplementary Table 7. Working life expectancy at age ≥50 years among all ELSA participants by sex and occupational position (n=9,649).

	Men									Women								
	High			Intermediate			Low			High			Intermediate			Low		
**Behaviour-related health risk factors**	**WLE**	**95% CI**		**WLE**	**95% CI**		**WLE**	**95% CI**		**WLE**	**95% CI**		**WLE**	**95% CI**		**WLE**	**95% CI**	
Number of risk factors																		
0	10.85	10.36	11.40	11.82	11.23	12.44	10.22	9.66	10.70	9.19	8.70	9.67	10.09	9.49	10.60	8.63	8.19	9.08
1	10.49	10.07	10.91	11.43	10.82	12.01	9.83	9.35	10.31	8.86	8.39	9.28	9.74	9.23	10.22	8.29	7.88	8.70
≥2	9.41	8.91	9.98	10.22	9.57	10.89	8.86	8.24	9.40	7.93	7.38	8.58	8.77	8.07	9.33	7.50	6.95	8.02
Obesity																		
No	10.54	10.13	10.94	11.48	10.94	12.04	9.87	9.45	10.31	8.96	8.54	9.37	9.87	9.43	10.32	9.87	9.45	10.31
Yes	9.80	9.24	10.36	10.62	9.93	11.20	9.24	8.73	9.80	8.37	7.81	8.92	9.18	8.61	9.78	9.24	8.73	9.80
Heavy drinking*																		
No	10.32	9.83	10.78	11.24	10.68	11.78	9.66	9.24	10.10	10.32	9.83	10.78	11.24	10.68	11.78	9.66	9.24	10.10
Yes	10.40	9.94	10.89	11.33	10.72	12.01	9.73	9.21	10.27	10.40	9.94	10.89	11.33	10.72	12.01	9.73	9.21	10.27
Current smoking																		
No	10.55	10.15	10.97	11.48	10.98	11.99	9.96	9.50	10.41	8.96	8.57	9.40	9.85	9.39	10.32	9.96	9.50	10.41
Yes	9.65	9.11	10.22	10.51	9.84	11.18	9.12	8.52	9.62	8.19	7.70	8.77	9.04	8.51	9.62	9.12	8.52	9.62
Low physical activity																		
No	10.50	10.08	10.90	11.51	11.02	12.03	9.91	9.51	10.33	8.95	8.52	9.40	9.86	9.41	10.37	9.91	9.51	10.33
Yes	8.79	7.46	9.58	9.63	7.83	10.71	8.30	7.12	9.13	7.52	6.56	8.37	8.32	7.20	9.13	8.30	7.12	9.13

Notes: * In ELSA high frequency of alcohol consumption and in FPS heavy alcohol consumption. WLE=working life expectancy
